# Microdose jet injection reveals a tissue-dependent retention–expression window for carrier-free nucleic acid delivery

**DOI:** 10.1080/10717544.2026.2738976

**Published:** 2026-10-30

**Authors:** Kazuhiro Terai, Ryusho Kariya, Hiromi Ogata-Aoki, Hiromitsu Iga, Satoshi Sakamoto, Seiji Sakano, Seiji Okada

**Affiliations:** a One Time Energy Collaborative Laboratory, Institute of Industrial Nanomaterials, Kumamoto University, Kumamoto, Japan; b Division of Hematopoiesis, Joint Research Center for Human Retrovirus Infection, Kumamoto University, Kumamoto, Japan; c Research Center & Life Science SBU, Tokyo Head Office, Daicel Corporation, Tokyo, Japan; d Laboratory of Molecular Cellular Biology, School of Pharmaceutical Sciences, Kobe Gakuin University, Kobe, Japan

**Keywords:** Microdose jet injection, needle-free delivery, nucleic acid delivery, local retention, tissue mechanics

## Abstract

Efficient local nucleic acid delivery requires not only access to target cells but also retention of the administered payload. Yet whether increasing physical delivery input improves or compromises functional delivery remains poorly understood. Here, we show that microdose jet injection operates within a tissue-dependent retention–expression window that can be interpreted in terms of physical jet input. Using the Actranza Lab needle-free system, sub-microlitre to low-microlitre jet outputs were calibrated and characterised by high-speed imaging, defining a kinetic-energy range of approximately 0.38–18.5 mJ. Delivery outcomes were evaluated in mouse spleen, liver, and skin using dye-retention analysis and luciferase expression. In spleen and liver, low-volume jets were associated with local retention and high volume-normalised luciferase expression, whereas increasing jet input progressively increased retention failure and reduced expression efficiency despite larger administered DNA amounts. In contrast, skin maintained local retention over a substantially broader input range, and total luciferase expression showed a significant positive association with injection volume. At the same 1-µL splenic administration volume, Actranza Lab produced approximately 34-fold greater luciferase activity than a 30-G Hamilton microsyringe. Cross-tissue integration of retention-failure frequency and volume-normalised expression revealed narrower operational windows in spleen and liver and a broader window in skin. These findings show that successful carrier-free jet delivery is determined not by nominal dose alone, but by the interaction between physical jet input and tissue retention. This retention–expression framework provides a practical basis for tissue-specific optimisation of needle-free nucleic acid delivery.

## Article highlights

- Microdose jet injection reveals a tissue-dependent retention–expression window.
- Spleen and liver show progressive retention failure as jet input increases.
- Low-volume retained delivery yields high volume-normalised expression in parenchymal tissues.
- Skin supports broad local retention and a positive volume–expression relationship.
- Tissue-specific retention may guide optimisation of carrier-free nucleic acid delivery.

## Graphical abstract

**Figure:**
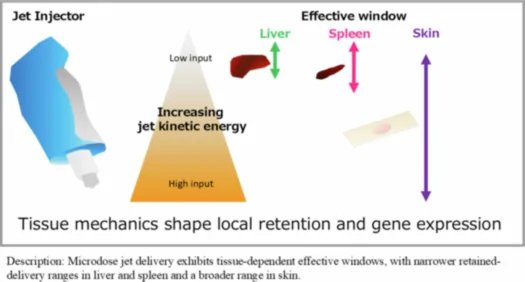

## 1. Introduction

Local delivery devices must operate within mechanically heterogeneous biological environments in which tissue properties can influence transport, retention, and intracellular uptake of administered therapeutics. Although advances in nucleic acid therapeutics have expanded the clinical potential of localised gene modulation (Langer and Tirrell 2004; Mitragotri et al. 2014), the physical constraints imposed by tissue characteristics on delivery-device performance remain insufficiently characterised. Consequently, the design of local delivery systems is often guided by empirical parameter selection rather than predictive understanding of device–tissue interactions. Because naked nucleic acids are susceptible to extracellular degradation, successful carrier-free delivery requires not only access to target cells but also sufficient local retention of the administered payload (Probst et al. 2006).

A variety of local delivery approaches, including needle-based injection, microneedles, and needle-free systems, have been developed to improve spatial control and tissue penetration of therapeutics (Prausnitz 2004; Kim et al. 2012). Among these, needle-free jet injection represents a distinctive physical delivery modality capable of dispersing liquid payloads within tissue compartments at high velocity while avoiding the limitations associated with conventional needle administration (Schramm-Baxter and Mitragotri 2004; Mitragotri 2006; Sonoda et al. 2025). The Actranza Lab system employs a high-pressure dual-ignition mechanism to generate high-velocity liquid jets (Miyazaki et al. 2019, 2021b). Prior studies have demonstrated that jet-mediated delivery can induce robust biological responses using naked plasmid DNA and messenger RNA without reliance on carrier systems (Chang et al. 2019; Abbasi et al. 2024; Tai et al. 2024; Terai et al. 2026; Miyazaki et al. 2021a), highlighting its potential as a carrier-free nucleic acid delivery strategy. However, the tissue-dependent conditions that determine whether jet-mediated administration results in functional delivery or payload loss remain poorly defined.

Biological tissues exhibit substantial variation in elastic modulus, structural organisation, and resistance to mechanical deformation, all of which may influence the outcome of high-velocity fluid injection (Discher et al. 2005; Engler et al. 2006; Chatelin et al. 2011; Girard et al. 2016; Song et al. 2022). Parenchymal organs such as the spleen and liver have structural architectures that may be more susceptible to penetration or disruption under localised mechanical loading (Chatelin et al. 2011; Girard et al. 2016; Song et al. 2022), whereas fibrous dermal tissue can accommodate substantial deformation while maintaining structural integrity (Song et al. 2022). These differences suggest that identical delivery conditions may produce distinct patterns of fluid retention, penetration, and functional delivery depending on the target tissue.

From a delivery-science perspective, successful local administration requires a balance between physical input sufficient to promote tissue entry and cellular interaction and preservation of local payload retention. Insufficient input may limit effective interaction between the delivered material and target tissue, whereas excessive input may create penetration pathways or leakage that reduce local exposure despite an increase in nominal administered dose. This suggests the existence of a tissue-dependent retention–expression window in which physical delivery remains functionally effective while local retention is preserved. Although recent advances in local delivery have explored approaches to improve tissue penetration and retention across different biological environments (Prausnitz 2004; Kim et al. 2012; Mitragotri et al. 2014), experimental evidence directly linking jet input, tissue retention, and functional gene expression across different tissues remains limited.

Here, we tested this concept by integrating sub-microlitre dose calibration, high-speed characterisation of jet velocity and kinetic energy, visible retention and penetration mapping, and in vivo luciferase expression across mouse spleen, liver, and skin. We further compared microdose jet delivery with conventional needle administration and integrated retention-failure frequency with volume-normalised expression to define tissue-specific delivery-response profiles. We aimed to determine whether increasing jet input continuously improves functional delivery or instead produces tissue-dependent transitions from retained delivery to payload loss. The resulting retention–expression framework provides a basis for tissue-specific selection of physical delivery parameters for carrier-free nucleic acid delivery.

## 2. Materials and methods

### 2.1. Animals and ethical approval

All animal procedures were approved by the Animal Experiment Review Committee of the Animal Reproduction Research Institute, Ibaraki, Japan (animal experiment protocol approval No. 5-27; experimental protocol No. 20230802-04; experimental code No. MDD-M-T-ETC20021-24) and were conducted in accordance with institutional guidelines and the ARRIVE guidelines. Under the institutional animal experiment approval system used for this study, these approval and protocol identifiers were issued for the approved animal experiments; no separate personal or institutional animal license number was issued. Male ICR mice aged 7–8 weeks were used in this study. Animals were housed under standard laboratory conditions with free access to food and water. Environmental enrichment was not specifically recorded. All surgical exposure and injection procedures were performed under isoflurane anaesthesia, and efforts were made to minimise pain and distress throughout the experiments. Animals were monitored during and after the procedures, and humane endpoints were applied if severe distress, marked bleeding, or abnormal recovery from anaesthesia was observed. No unexpected adverse events occurred during the experiments. At the experimental endpoints, animals were euthanized under deep isoflurane anaesthesia before tissue collection. To reduce unwanted variability, experiments were performed using mice of the same sex and similar age, predefined injection volumes, standardised injection positioning, and identical tissue-collection time points.

### 2.2. Jet injection system and volume calibration

Jet injections were performed using the Actranza® Lab Mouse System (Daicel), which employs a dual-ignition pressurisation mechanism to generate high-velocity liquid jets (Miyazaki et al. 2019). Effective ejection volumes were calibrated gravimetrically using malachite green solution, enabling establishment of a reproducible sub-microlitre microdose injection regime. Defined low-volume conditions (1.3 µL and 5.3 µL) were generated by placing droplets (1 µL or 5 µL) onto the nozzle tip and re-aspirating them into the chamber, extending previously reported container-handling procedures (Miyazaki et al. 2019). All injection volumes were confirmed using a microbalance and the measured solution density (1.0018 g/mL).

### 2.3. Jet velocity and kinetic energy measurement

Jet dynamics were captured using a high-speed camera system (Photron SA-X2) operating at 20,000 frames per second within a transparent tube containing a 1-mm grid reference. Jet velocity was calculated from frame-to-frame displacement measurements, and kinetic energy was estimated using

E=½mv2

assuming a liquid density of 1.0 g/cm^3^. These measurements provided the physical input parameters used to contextualise tissue responses.

### 2.4. Surgical exposure of spleen and liver

For splenic injection, a ~1-cm left lateral abdominal incision was performed, and the spleen was gently exteriorised while maintaining capsular integrity. For hepatic injection, a midline incision was made to expose the median liver lobe, which was stabilised using sterile cotton applicators during jet administration to minimise tissue displacement.

### 2.5. Intradermal injection procedures

For intradermal experiments, dorsal skin was shaved before injection. Jet injections were applied perpendicular to the skin surface. Malachite green solution (1% w/v) was used to confirm dermal deposition and assess potential subcutaneous leakage, consistent with prior observations of dermal confinement following jet delivery (Miyazaki et al. 2019).

### 2.6. Plasmid preparation and administration

A luciferase-encoding plasmid (pGL4.13-luc2; Promega) was prepared at 1 mg/mL in phosphate-buffered saline. Parenchymal organ injections were performed at volumes of 0.3, 1.3, 3.3, and 5.3 µL, whereas intradermal injections were conducted across a range of 0.3–20.3 µL. For comparison, needle-syringe administration was performed using a 30-G Hamilton microsyringe.

### 2.7. Tissue collection and luciferase quantification

Twenty-four hours after injection, the whole spleen, a liver tissue block of approximately 1 cm × 1 cm centred on the injection site (Supplementary Fig. 1), or an 8-mm skin biopsy (Biopsy puncher, BP-80F, KAI Corporation, Tokyo, Japan) centred on the injection site was collected. Each sample was minced in an appropriate volume of Passive Lysis Buffer (Promega), subjected to a single freeze–thaw cycle, and centrifuged at 10,000 × g for 10 min. Supernatants were diluted with Passive Lysis Buffer as necessary to bring the luminescence signal within the measurable range of the luminometer. Aliquots of 20 µL were mixed with luciferase substrate, and luminescence was measured for 10 s. Measured RLU values were corrected for the corresponding dilution factor to calculate total RLU for each collected tissue sample. Background luminescence was determined using the corresponding tissue from animals not receiving plasmid DNA and was subtracted from the measured values. Where indicated, total RLU was further normalised to the administered injection volume.

### 2.8. Assessment of tissue penetration

Penetration following malachite green injection was evaluated by dissection of injection sites and visual assessment of dye distribution. Penetration was classified as absent, partial, or complete based on whether dye traversed tissue thickness. Observations were interpreted in the context of previously reported mechanical characteristics of soft organs (Girard et al. 2016; Song et al. 2022). For the descriptive retention-failure analysis, an injection was classified as a retention failure when visible dye penetration through the tissue or loss of local dye confinement was observed.

### 2.9. Study design, bias control, and statistical analysis

This study was designed as an exploratory parameter-mapping study to evaluate the relationships among microdose jet input, tissue retention or penetration, and luciferase expression across mouse spleen, liver, and skin. The experimental unit was an individual animal or tissue sample, as indicated in the corresponding figure legends. Injection volumes and tissue types were predefined before the experiments. No formal a priori sample-size calculation was performed; sample sizes were selected based on feasibility and previous jet-injection studies and are reported in the figure legends. No predefined inclusion or exclusion criteria were applied, and no animals or data points were excluded from the analyses. Randomisation and blinding were not performed. To reduce unwanted variability, experiments were conducted using mice of the same strain, sex, and age range, standardised injection positioning, predefined injection volumes, and identical tissue-collection time points.

Outcome measures included ejection volume, jet velocity, calculated kinetic energy, visible dye retention or penetration, total luciferase activity, and volume-normalised luciferase activity. Data are presented as mean ± SD unless otherwise stated. Luciferase data were analysed using the original, untransformed values. For comparisons among injection-volume groups within each tissue, ordinary one-way analysis of variance (ANOVA) followed by Tukey’s multiple-comparisons test was used for both total and volume-normalised luciferase activity. For skin, the relationship between injection volume and total luciferase activity was additionally evaluated using Spearman rank correlation based on the mean luciferase value at each of the six tested injection volumes. Retention-failure outcomes in the dye studies were summarised descriptively as the number of failures among three injections at each volume condition. For the comparison of splenic luciferase expression following 1-µL administration by a 30-G Hamilton microsyringe or Actranza Lab, a two-sided Welch’s unpaired t-test was used. Statistical significance was defined as *p* < 0.05. Statistical analyses were performed using GraphPad Prism version 10.3.1 (GraphPad Software, Boston, MA, USA).

## 3. Results

### 3.1. Calibration of microdose jet output defines the delivery input range

To define the physical input range for microdose jet delivery, we first calibrated the ejection volume of the Actranza Lab injector by gravimetric analysis (Figure 1). Manual emptying of the nozzle chamber reproducibly left a residual dead volume of approximately 1.4 µL. When the container retaining this residual volume was mounted on the Actranza Lab Mouse system and actuated, approximately 0.3 µL of liquid was additionally discharged, defining a reproducible sub-microlitre microdose jet. Additional low-volume conditions were prepared by aspirating defined droplets into the chamber, enabling controlled delivery in the sub-microlitre to low-microlitre range.

**Figure 1.**
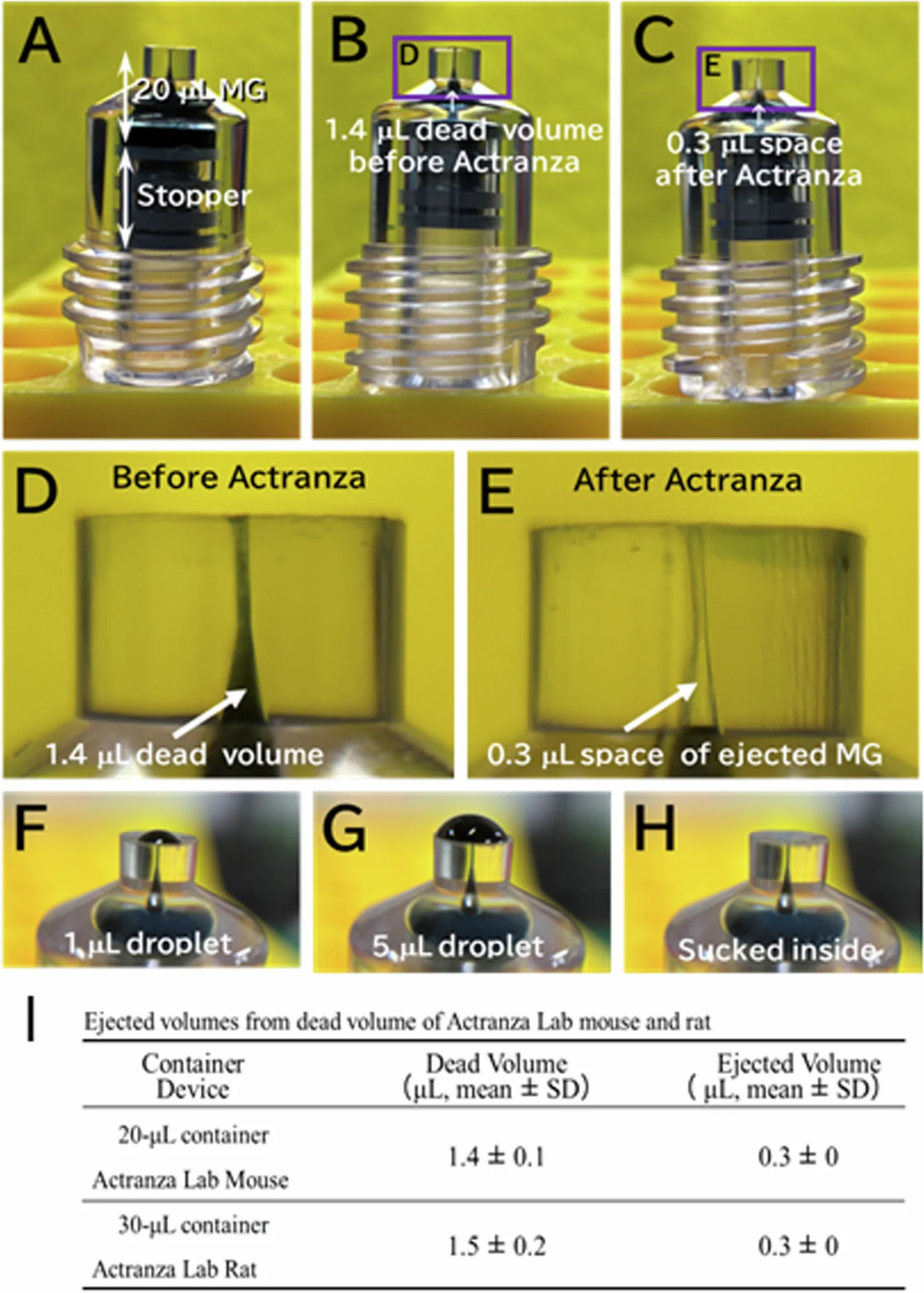
Dead-volume characterisation and preparation of defined microdose jet volumes using Actranza Lab containers. (A–E) Representative images showing malachite green (MG) solution in a standard 20-µL Actranza Lab container before and after manual ejection, and after subsequent jet actuation with the Actranza Lab Mouse system. Manual ejection left a residual dead volume of approximately 1.4 µL, and jet actuation discharged approximately 0.3 µL from this residual volume. (F–H) Preparation of defined low-microlitre jet volumes. After manual ejection, 1 µL or 5 µL of MG solution was placed on the container surface and re-aspirated into the chamber to prepare the 1.3-µL or 5.3-µL settings, respectively. (I) Quantification of residual dead volume and jet-ejected volume for the 20-µL and 30-µL Actranza Lab containers. Data are shown as mean ± SD; *n* = 5 per group.

High-speed imaging was then used to characterise the corresponding jet velocity and kinetic energy (Figure 2). Across the tested 0.3–10.3 µL range, jet velocities were approximately 50–80 m/s, corresponding to kinetic energies of 0.38–18.5 mJ. These measurements established the relationship between ejection volume and mechanical input, providing the physical parameter space used to evaluate tissue retention and gene-expression outcomes. Velocity measurements shown in Figure 2 were obtained once per volume condition and were used as representative system-output measurements.

**Figure 2.**
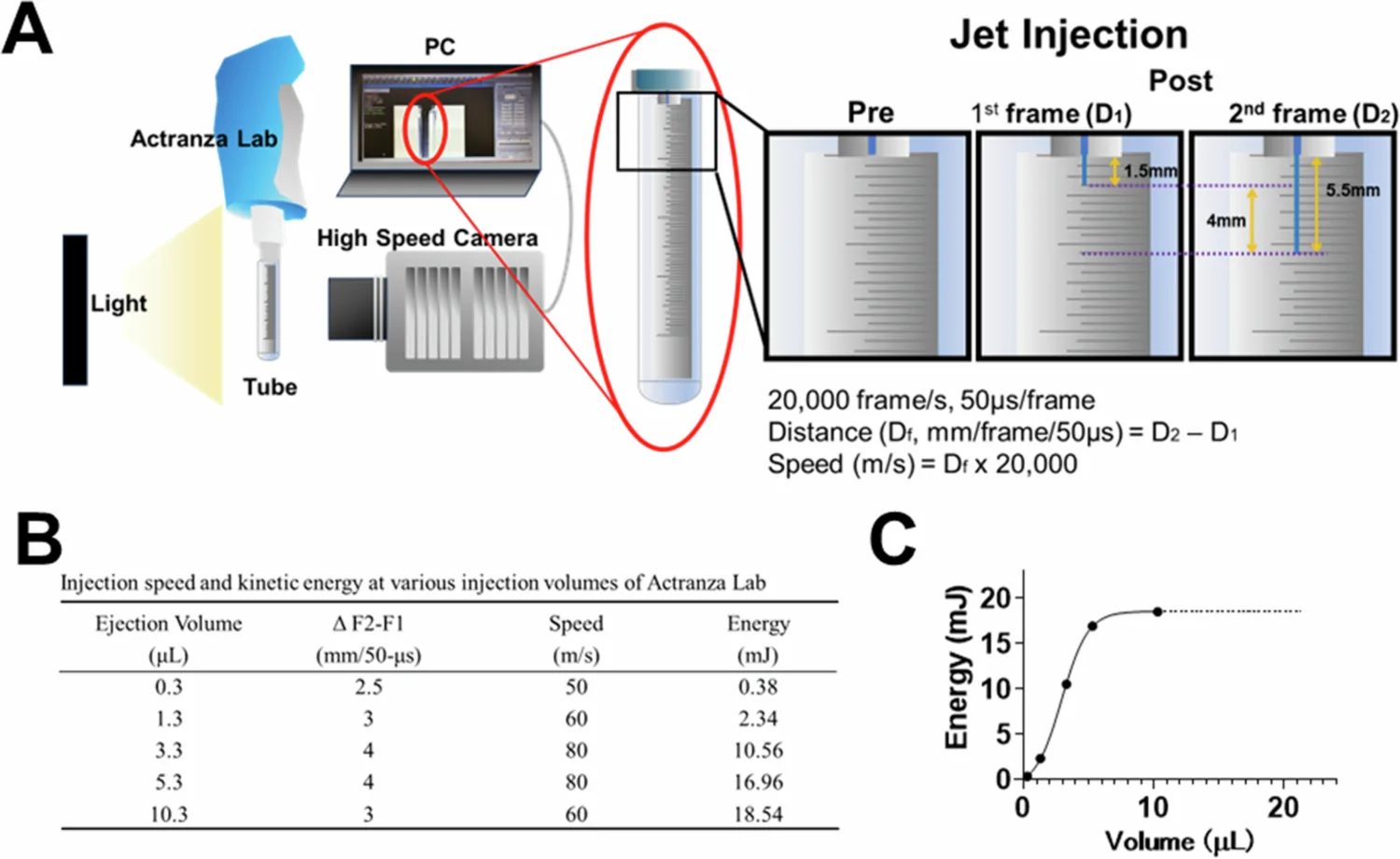
High-speed imaging and kinetic characterisation of microdose jet injection. (A) High-speed camera system used to capture Actranza Lab jet ejection. Image acquisition was synchronised with Actranza Lab Mouse actuation and performed at 20,000 frames/s with a 50-µs frame interval. (B) Jet velocity and calculated kinetic energy at defined ejection volumes of 0.3, 1.3, 3.3, 5.3, and 10.3 µL. Jet velocity was measured from high-speed images, and kinetic energy was calculated as described in the Materials and Methods. Measurements were performed once per volume condition and are presented as representative system-output measurements. (C) Four-parameter logistic fitting of the energy–volume relationship. Because the curve approached a plateau between 5.3 and 10.3 µL, values beyond 10.3 µL are shown as an extrapolated dotted extension.

### 3.2. Parenchymal tissues show volume-dependent retention failure during microdose jet injection

We next examined how microdose jets interacted with mouse spleen and liver using malachite green (MG) as a visible marker of local fluid retention and tissue penetration (Figure 3F). In the spleen, no retention failure was observed at 0.3 or 1.3 µL (0/3 injections at each volume), whereas failure was observed in 1/3 injections at 3.3 µL and in 3/3 injections at 5.3 µL. In the liver, retention failure occurred at lower input, with frequencies of 0/3, 1/3, 2/3, and 3/3 injections at 0.3, 1.3, 3.3, and 5.3 µL, respectively. At higher tested volumes, penetration or loss of locally confined dye was readily observed in both organs (Figure 3F). These observations demonstrate a progressive loss of local fluid retention as jet input increased, with the liver showing an earlier transition than the spleen. Thus, the range over which injected fluid remained locally confined differed between the two parenchymal tissues.

**Figure 3.**
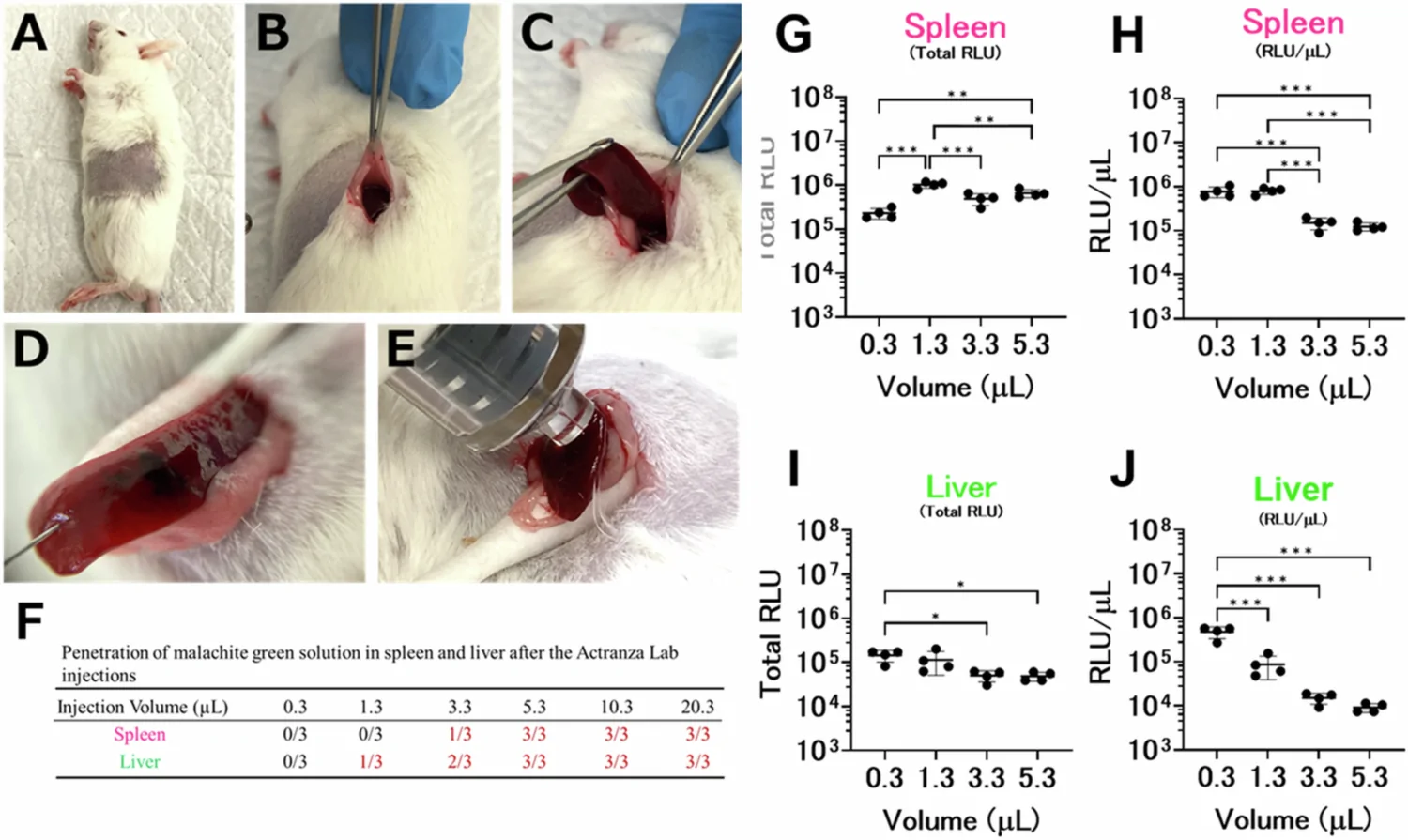
Microdose jet injection into mouse spleen and liver: tissue retention, penetration, and luciferase expression. (A–C) Surgical exposure of the mouse spleen. The left lateral abdomen was shaved, a small incision was made, and the spleen was gently exteriorised while preserving the organ surface and vasculature. (D, E) Injection positioning for needle-syringe and Actranza Lab jet administration. Needle-syringe injection was performed using a 30-G needle inserted horizontally, whereas jet injection was performed by placing the nozzle vertically against the organ surface with gentle stabilisation. (F) Tissue-penetration assessment after Actranza Lab injection of 1% malachite green (MG) into spleen or liver at 0.3, 1.3, 3.3, 5.3, 10.3, and 20.3 µL. Penetration was evaluated by visible dye leakage on the dorsal side of each organ; *n* = 3 per volume and tissue. (G–J) Luciferase expression 24 h after pLuc administration using the Actranza Lab Mouse system. pLuc solution was injected into the spleen or liver at 0.3, 1.3, 3.3, or 5.3 µL. Total luciferase activity and volume-normalised activity are shown for spleen (G, H) and liver (I, J), respectively. Individual values and mean ± SD are shown; *n* = 4 per group. Data are displayed on logarithmic y-axes. Statistical significance was assessed by ordinary one-way ANOVA followed by Tukey’s multiple-comparisons test. Only statistically significant pairwise comparisons are indicated. *p < 0.05, **p < 0.01, ***p < 0.001.

### 3.3. Luciferase expression efficiency is highest within the retained microdose range

To determine whether these retention patterns were associated with functional delivery, a luciferase plasmid was administered to the spleen and liver using the same microdose jet conditions (Figure 3G–J). In the spleen, total luciferase activity was highest at 0.3–1.3 µL and decreased at higher injection volumes, with significant pairwise differences among injection-volume groups (Figure 3G). Volume-normalised luciferase activity showed an even clearer decline as injection volume increased (Figure 3H), indicating that the amount of functional expression generated per unit volume was greatest within the low-volume retained range. A similar pattern was observed in the liver. Total luciferase activity was greatest at the lowest tested volume and was significantly lower at selected higher-volume conditions (Figure 3I). Volume-normalised activity decreased markedly with increasing injection volume, with significant differences between 0.3 µL and each of the higher-volume groups (Figure 3J). Thus, increasing the administered plasmid volume did not produce a proportional increase in functional gene expression in either spleen or liver.

The functional advantage of jet delivery was further supported by comparison with conventional needle administration. Following 1-µL splenic administration, Actranza Lab produced approximately 34-fold greater luciferase activity than a 30-G Hamilton microsyringe (622,200 vs 18,363 mean RLU; *p* = 0.0148, two-sided Welch’s unpaired t-test; Supplementary Fig. 2). Together, these findings indicate that functional delivery in spleen and liver is favoured within the low-volume range in which local retention is maintained, rather than simply by increasing the nominal DNA dose.

### 3.4. Skin supports broad local retention and a positive volume–expression relationship

In contrast to spleen and liver, dermal tissue maintained local dye confinement over a substantially broader injection range (Figure 4A–D). Intradermal MG administration produced visible wheal formation from 1.3 to 20.3 µL, and postmortem dissection showed confinement of the dye within the dermal injection site without detectable penetration into the underlying muscle (Figure 4A–C). At 0.3 µL, successful intradermal deposition could not be confirmed reliably; therefore, this condition was classified as uncertain rather than as retained or failed. No retention failure was observed at 1.3–20.3 µL (0/3 injections at each tested volume; Figure 4D).

**Figure 4.**
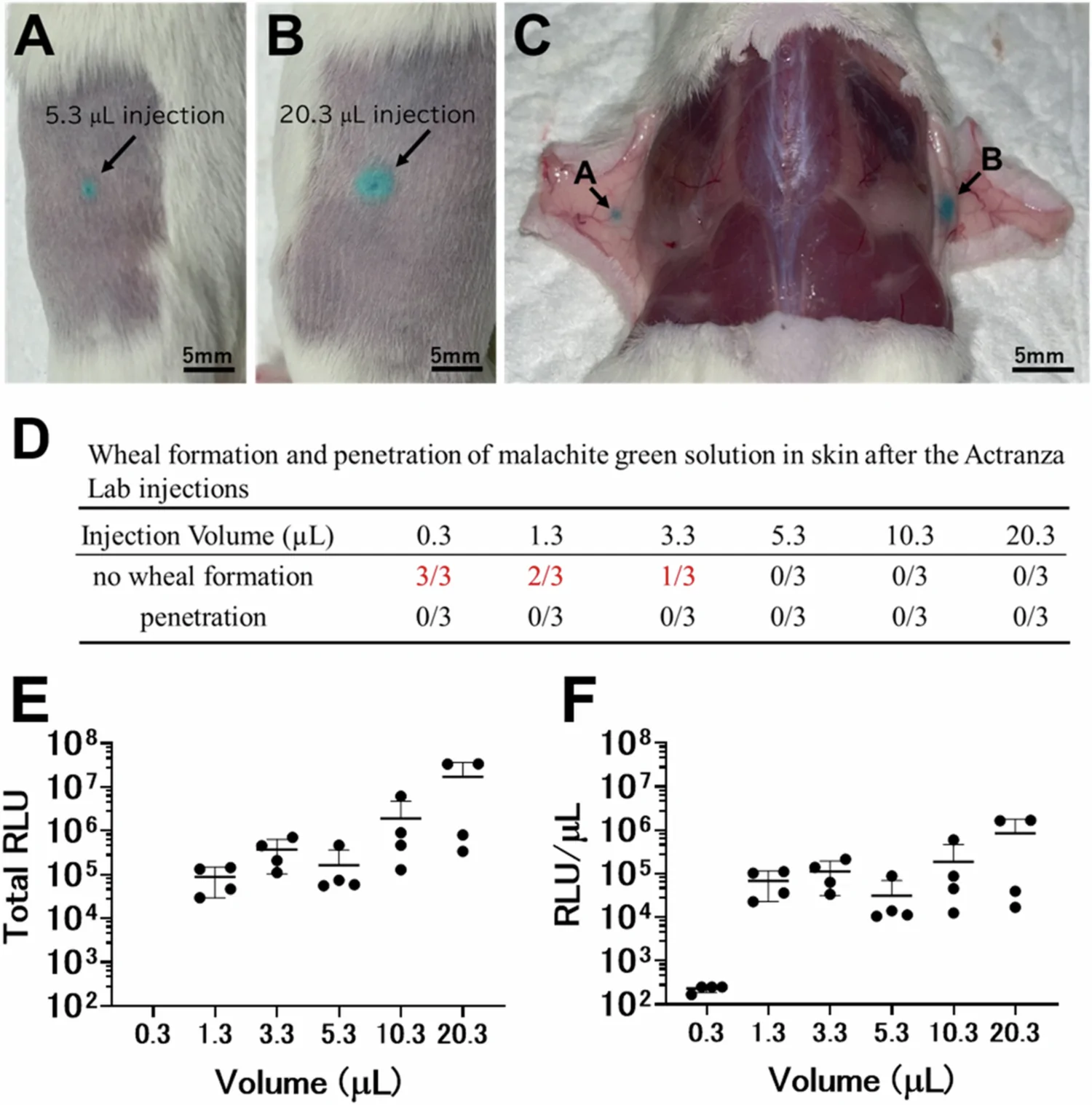
Intradermal microdose jet injection supports broad dye confinement and a positive volume–expression relationship in mouse skin. (A–C) Representative outcomes of intradermal Actranza Lab jet injection. Injection of 1% MG produced volume-dependent blue wheal formation at the skin surface, and postmortem dissection confirmed dye confinement within the dermal injection site without detectable penetration into the underlying muscle layer. Scale bar = 5 mm. (D) Assessment of wheal formation and subcutaneous leakage after intradermal injection of 1% MG at 0.3, 1.3, 3.3, 5.3, 10.3, and 20.3 µL; *n* = 3 per volume. (E, F) Luciferase expression 24 h after intradermal pLuc administration using the Actranza Lab Mouse system. Total intradermal luminescence and volume-normalised luminescence are shown. Individual values and mean ± SD are shown; *n* = 4 per group. Data are displayed on logarithmic y-axes. Lower error bars are automatically omitted where applicable. Statistical significance among injection-volume groups was assessed by ordinary one-way ANOVA followed by Tukey’s multiple-comparisons test. No statistically significant pairwise differences were detected. For total luciferase activity, the association with injection volume was additionally assessed by Spearman rank correlation using the mean value at each injection volume (r = 0.9429, *p* = 0.0167).

Luciferase expression was detected across the tested intradermal range (Figure 4E, F). Although ordinary one-way ANOVA followed by Tukey’s multiple-comparisons test did not identify significant pairwise differences among injection-volume groups for either total or volume-normalised luciferase activity, total luciferase activity showed a significant positive association with injection volume when the mean value at each of the six tested volumes was analysed by Spearman rank correlation (r = 0.9429, *p* = 0.0167; Figure 4E). In contrast, volume-normalised luciferase activity did not show the pronounced efficiency collapse observed in spleen and liver at higher injection volumes (Figure 4F). These findings indicate that skin accommodates a substantially broader range of jet inputs while maintaining local retention, with total functional expression increasing across the tested volume range.

### 3.5. Integrated retention–expression mapping reveals tissue-dependent delivery windows

To directly compare delivery behaviour across tissues, retention-failure outcomes and volume-normalised luciferase expression were integrated across spleen, liver, and skin (Figure 5). Mean RLU/µL values were normalised to the maximum mean value within each tissue and expressed as the percentage of the tissue-specific maximum, enabling comparison of response profiles despite differences in absolute expression magnitude (Figure 5A). Tissue-specific retention–expression profiles were then mapped against the experimentally observed retention states (Figure 5B–D), and retention-failure frequencies were summarised across all tested tissue–volume combinations (Figure 5E).

**Figure 5.**
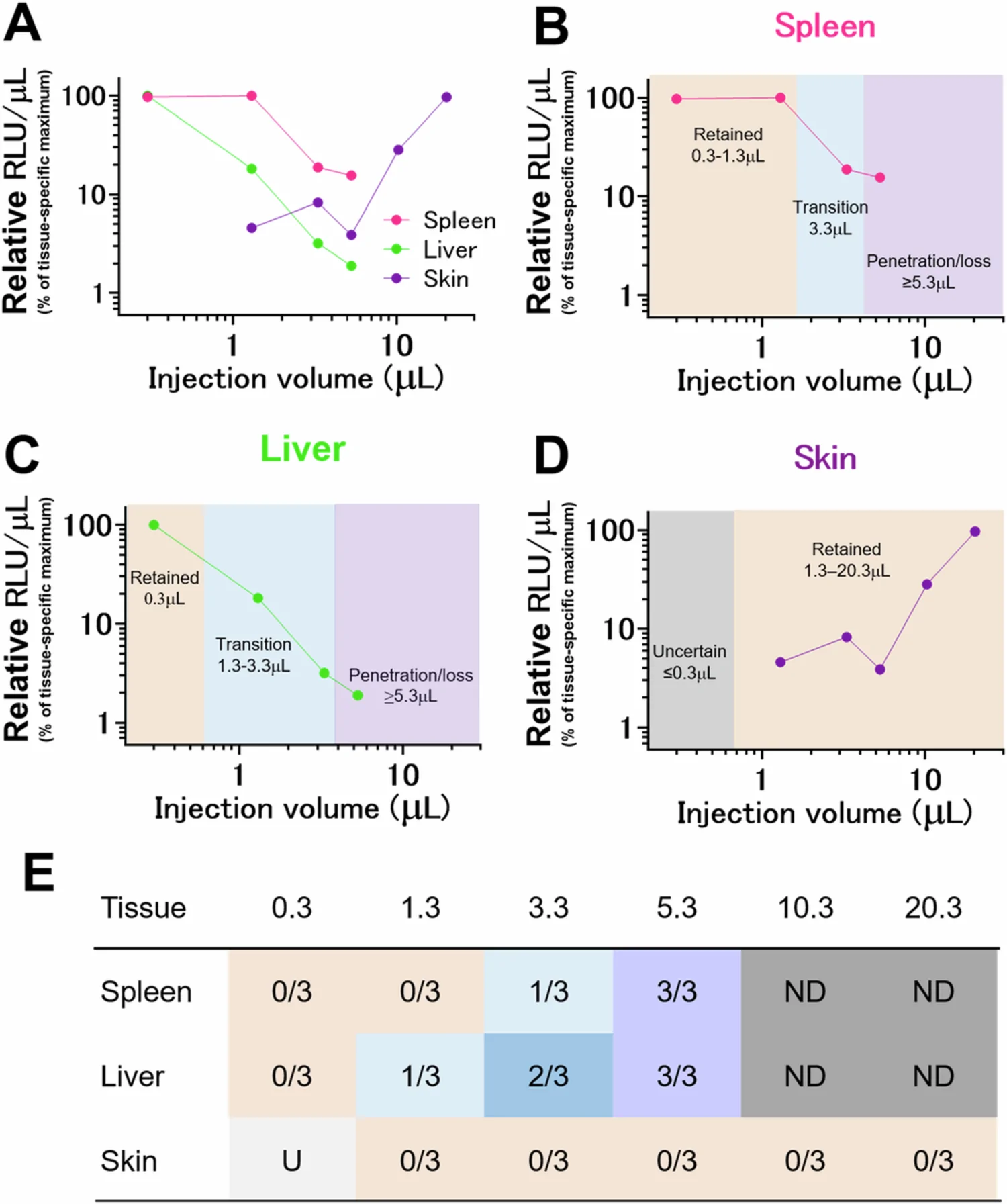
Integrated comparison of tissue-dependent retention and expression profiles following microdose jet injection. (A) Cross-tissue comparison of volume-normalised luciferase expression in spleen, liver, and skin. Mean RLU/µL values from the datasets shown in Figures 3 and 4 were normalised to the maximum mean value within each tissue and expressed as a percentage of the tissue-specific maximum. Lines connect the mean values at the tested injection volumes to facilitate comparison of tissue-specific response profiles. (B–D) Integrated retention–expression profiles for spleen (B), liver (C), and skin (D). Background shading indicates delivery outcome categories derived from the malachite green retention/penetration assessment. Retained indicates conditions with no observed retention failure (0/3), Transition indicates conditions with partial retention failure (1/3–2/3), and Penetration/loss indicates conditions with retention failure in all tested injections (3/3). For skin, the 0.3-µL condition was classified as Uncertain because successful intradermal delivery could not be confirmed, whereas retention was observed over the 1.3–20.3-µL range. The overlaid lines show the corresponding tissue-specific relative RLU/µL profiles presented in (A). (E) Experimental retention-failure map summarising the observed frequency of retention failure at each tested injection volume. Values indicate the number of injections showing retention failure among three injections. Increasing colour intensity denotes increasing failure frequency. U, uncertain; ND, not determined.

Spleen and liver showed narrow high-efficiency regions at low injection volumes, followed by increasing retention failure and reduced normalised expression as jet input increased (Figure 5B, C, E). The transition occurred earlier in liver than in spleen, consistent with the respective MG retention-failure frequencies. In contrast, skin maintained retention from 1.3 to 20.3 µL and exhibited a substantially broader expression profile without the abrupt efficiency loss observed in the parenchymal organs (Figure 5A, D, E). The integrated analysis therefore identifies distinct tissue-dependent retention–expression windows, with narrower operational ranges in spleen and liver and a substantially broader range in skin.

## 4. Discussion

This study identifies a tissue-dependent retention–expression window for microdose jet-mediated nucleic acid delivery. By integrating calibrated microdose ejection, high-speed characterisation of jet output, visible retention and penetration outcomes, and in vivo luciferase expression, we found that increasing administered volume did not uniformly increase functional delivery. In spleen and liver, low-volume conditions were associated with high volume-normalised expression, whereas increasing jet input was accompanied by progressive retention failure and reduced expression efficiency. In contrast, skin maintained local retention across a substantially broader range of injection volumes and showed a significant positive association between injection volume and total luciferase expression. Cross-tissue integration further revealed distinct delivery-response profiles, with narrower operational windows in spleen and liver and a broader window in skin. These findings indicate that functional delivery depends not on nominal dose alone, but on whether physical input remains compatible with local payload retention.

The retention–expression window provides a delivery-oriented framework for interpreting the relationship between jet input and biological output. Within a retained microdose range, localised shear, interstitial deformation, and fluid dispersion may increase opportunities for cellular interaction and uptake, consistent with previous reports suggesting shear- or endocytosis-associated mechanisms in jet-mediated delivery (Miyazaki et al. 2021a; Terai et al. 2025). However, when jet input exceeds the structural tolerance of the target tissue, penetration pathways may form and promote loss of the administered solution from the intended site. Under such conditions, increasing the nominal DNA dose does not necessarily increase effective local delivery because payload residence within the tissue becomes compromised. The present MG-distribution data support this interpretation at the level of macroscopic fluid behaviour, although they do not directly demonstrate the intratissue distribution or retained mass of plasmid DNA.

A major implication of this study is that optimisation of physical delivery systems should not rely on injection volume alone. In spleen and liver, increasing injection volume increased the amount of administered DNA but was accompanied by a transition toward retention failure and lower volume-normalised expression. Because injection volume in the present system was also associated with changes in jet kinetic energy, delivered volume and physical input are intrinsically linked within the tested parameter range. Importantly, however, the transition occurred at different volumes in spleen and liver, indicating that a single universal input threshold cannot be assigned across tissues. Jet input, tissue-specific retention capacity, and functional output should therefore be considered together when selecting delivery conditions. This perspective may be particularly important for carrier-free nucleic acid delivery because naked nucleic acids are susceptible to extracellular degradation and may require rapid cellular access within a confined local environment (Probst et al. 2006).

The contrasting behaviour of skin further emphasises the importance of tissue-specific parameter selection. Unlike spleen and liver, skin maintained visible local dye confinement from 1.3 to 20.3 µL. Although pairwise comparisons among individual injection-volume groups did not reach statistical significance, total luciferase activity showed a significant positive association with injection volume. Moreover, volume-normalised expression did not show the pronounced high-volume decline observed in the parenchymal organs. This broader operational range is consistent with the fibrous architecture and deformation tolerance of dermal tissue (Song et al. 2022), although mechanical properties were not measured directly in the present study. These findings are also consistent with previous reports showing robust intradermal expression following jet-mediated delivery of naked plasmid DNA or mRNA (Abbasi et al. 2024; Tai et al. 2024; Terai et al. 2026; Miyazaki et al. 2021a). More broadly, the results suggest that tissues differ substantially in the range of jet inputs over which local retention and functional expression can be maintained.

The comparison with conventional needle administration provides additional context for the functional efficiency of microdose jet delivery. At the same 1-µL splenic administration volume, Actranza Lab produced approximately 34-fold greater luciferase activity than a 30-G Hamilton microsyringe. Although this comparison was performed as a separate experiment and was limited to a single volume and tissue site, it indicates that the high expression observed at low jet input cannot be explained by administered DNA amount alone. Rather, the mode of physical delivery itself appears to contribute substantially to functional nucleic acid delivery. Together with the retention–expression analysis, these findings provide a practical rationale for tissue-informed optimisation of needle-free delivery systems. Rather than applying a single device setting across different anatomical targets, delivery parameters should be selected according to the retention behaviour and functional response of the intended tissue. The specific windows identified here should therefore be regarded as device-, payload-, and tissue-context-dependent rather than as universal parameter ranges.

This study has several limitations. First, the experiments were exploratory and used relatively small sample sizes, and no formal a priori sample-size calculation was performed. The statistical analyses should therefore be interpreted primarily as supporting the observed delivery-response patterns rather than as definitive estimates of effect size. Second, jet velocity and kinetic energy were characterised using one representative analysed jet per volume condition; repeated high-speed measurements will be required to quantify system-output variability more rigorously. Third, tissue retention was assessed using visible MG localisation and penetration as a surrogate for macroscopic fluid retention, rather than by direct measurement of retained plasmid mass or time-resolved intratissue distribution. Fourth, luciferase expression was evaluated at a single post-injection time point, and total RLU and volume-normalised RLU were used as functional output measures rather than normalisation to tissue protein content. Accordingly, these measurements describe local functional expression and expression per administered volume, but do not directly quantify transfection efficiency per unit tissue protein. Fifth, tissue mechanical properties were inferred from tissue architecture and previously reported biomechanical characteristics rather than measured directly in the experimental samples. Sixth, plasmid-mediated expression may also depend on vector regulatory sequences, CpG content, host-cell type, and innate immune responses. CpG-containing and CpG-free plasmid vectors can differ in inflammatory responses and the magnitude or persistence of transgene expression (Hyde et al. 2008). Because the same pGL4.13-luc2 construct was used across all injection conditions in the present study, plasmid sequence was held constant within the present comparisons; however, the quantitative retention–expression profiles observed here may not directly extrapolate to other plasmid constructs or tissue contexts. Finally, the needle comparison was limited to a single 1-µL splenic condition and should not be interpreted as a comprehensive comparison between jet and conventional injection across tissues or doses. Future studies incorporating larger cohorts, repeated jet-output measurements, direct payload quantification, quantitative tissue mechanics, multiple expression time points, and additional nucleic acid constructs will be required to refine the predictive value and generalisability of the retention–expression framework.

## 5. Conclusions

This study demonstrates that microdose jet-mediated nucleic acid delivery operates within a tissue-dependent retention–expression window. In spleen and liver, low-volume jet conditions were associated with local retention and high volume-normalised luciferase expression, whereas increasing jet input was accompanied by progressive retention failure and reduced expression efficiency despite larger administered DNA amounts. In contrast, skin maintained local retention over a substantially broader injection range and showed a significant positive association between injection volume and total luciferase expression. These findings indicate that nominal dose alone is insufficient to predict delivery performance and that effective carrier-free jet delivery depends on matching physical input to the retention behaviour of the target tissue. The retention–expression framework described here may therefore provide a practical basis for tissue-specific optimisation of needle-free nucleic acid delivery.

## Supplementary Material

Supplementary Material Suppl_Figure_1.tif

Supplementary Material Suppl_Figure_2_Hi-Resolution.tif

Supplementary Material Author_Checklist_Full_for_DD_R1.pdf

Supplementary Material High Lights for DD R1.docx

Supplementary Material Graphical Abstract R1 final.tif

Supplementary Material Legends for Supplementary Figs.docx

## Data Availability

The data that support the findings of this study are available from the corresponding author upon reasonable request.

## AI use statement

The authors used ChatGPT (OpenAI, GPT-5.5 Thinking) and DeepL to assist with language editing, translation support, and structural refinement of the manuscript. These tools were not used to generate original experimental data, perform statistical analyses, generate or manipulate images, or draw scientific conclusions. The graphical abstract was created by the authors using Microsoft PowerPoint without the use of AI-based image generation tools. All AI-assisted text was reviewed, revised, and approved by the authors, who take full responsibility for the manuscript's content.
