# Supplementary material for: Microdose jet injection reveals a tissue-dependent retention–expression window for carrier-free nucleic acid delivery
Source: Drug Deliv. 2026 Oct 30;33(1):2738976. doi: 10.1080/10717544.2026.2738976 (PMC13726303; doi:10.1080/10717544.2026.2738976)
Supplement: Supplementary file 1 [file IDRD_A_2738976_SM3433.pdf]

NOTE: Please save this file locally before filling in the table, DO NOT work on the file within your internet browser as changes will not be saved. Adobe Acrobat Reader (available free here) is recommended for completion.

The ARRIVE guidelines 2.0: author checklist

The ARRIVE Essential 10

These items are the basic minimum to include in a manuscript. Without this information, readers and reviewers cannot assess the reliability of the findings.

Item		Recommendation	Section/line number, or reason for not reporting
**Study design**	1	For each experiment, provide brief details of study design including:	
		a. The groups being compared, including control groups. If no control group has been used, the rationale should be stated.	Sections 2.6, 2.8, 2.9, and Results Sections 3.1–3.5. Predefined microdose jet injection volumes were compared across mouse spleen, liver, and skin. Needle-syringe administration was included as a quantitative comparison at the 1-µL splenic condition.
		b. The experimental unit (e.g. a single animal, litter, or cage of animals).	Section 2.9 and figure legends. The experimental unit was an individual animal or tissue sample, as indicated in the corresponding figure legends.
**Sample size**	2	a. Specify the exact number of experimental units allocated to each group, and the total number in each experiment. Also indicate the total number of animals used.	Section 2.9 and figure legends for Figs. 1–4 and Supplementary Fig. 2. Exact n values are reported in the corresponding figure legends.
		b. Explain how the sample size was decided. Provide details of any *a priori* sample size calculation, if done.	Section 2.9. No formal a priori sample-size calculation was performed; sample sizes were selected based on feasibility and previous jet-injection studies.
**Inclusion and exclusion criteria**	3	a. Describe any criteria used for including and excluding animals (or experimental units) during the experiment, and data points during the analysis. Specify if these criteria were established *a priori*. If no criteria were set, state this explicitly.	Section 2.9. No predefined inclusion or exclusion criteria were applied.
		b. For each experimental group, report any animals, experimental units or data points not included in the analysis and explain why. If there were no exclusions, state so.	Section 2.9. No animals, experimental units, or data points were excluded from the analyses.
		c. For each analysis, report the exact value of *n* in each experimental group.	Figure legends for Figs. 1–4 and Supplementary Fig. 2. Exact n values are reported in the corresponding figure legends.
**Randomisation**	4	a. State whether randomisation was used to allocate experimental units to control and treatment groups. If done, provide the method used to generate the randomisation sequence.	Section 2.9. Randomisation was not performed because injection volumes and tissue types were predefined in this exploratory parameter-mapping study.
		b. Describe the strategy used to minimise potential confounders such as the order of treatments and measurements, or animal/cage location. If confounders were not controlled, state this explicitly.	Sections 2.1 and 2.9. Potential variability was reduced by using mice of the same strain, sex, and age range, standardized injection positioning, predefined injection volumes, and identical tissue-collection time points.
**Blinding**	5	Describe who was aware of the group allocation at the different stages of the experiment (during the allocation, the conduct of the experiment, the outcome assessment, and the data analysis).	Section 2.9. Blinding was not performed. The study involved predefined injection conditions and outcome measures including ejection volume, jet velocity, dye retention or penetration, and luciferase activity.
**Outcome measures**	6	a. Clearly define all outcome measures assessed (e.g. cell death, molecular markers, or behavioural changes).	Sections 2.2, 2.3, 2.7, 2.8, 2.9, and Results Sections 3.1–3.5. Outcome measures included ejection volume, jet velocity, calculated kinetic energy, visible dye retention or penetration, total luciferase activity, and
		b. For hypothesis-testing studies, specify the primary outcome measure, i.e. the outcome measure that was used to determine the sample size.	Section 2.9. This was an exploratory parameter-mapping study, and no single primary outcome measure was used to determine sample size.
**Statistical methods**	7	a. Provide details of the statistical methods used for each analysis, including software used.	Section 2.9 and figure legends. Luciferase data were analyzed using the original, untransformed values. Ordinary one-way ANOVA followed by Tukey's multiple-comparisons test was used for
		b. Describe any methods used to assess whether the data met the assumptions of the statistical approach, and what was done if the assumptions were not met.	Section 2.9. Luciferase data were analyzed using the original, untransformed values. No separate formal tests of distributional assumptions were performed because of the small group sizes; statistical results were
**Experimental animals**	8	a. Provide species-appropriate details of the animals used, including species, strain and substrain, sex, age or developmental stage, and, if relevant, weight.	Section 2.1. Male ICR mice aged 7–8 weeks were used in this study.
		b. Provide further relevant information on the provenance of animals, health/immune status, genetic modification status, genotype, and any previous procedures.	Section 2.1. Animals were housed under standard laboratory conditions with free access to food and water. No genetic modification or previous procedures were involved.
**Experimental procedures**	9	For each experimental group, including controls, describe the procedures in enough detail to allow others to replicate them, including:	
		a. What was done, how it was done and what was used.	Sections 2.2–2.8 and figure legends for Figs. 1–4 and Supplementary Figs. 1 and 2. Procedures included jet injection system calibration, high-speed imaging, surgical
		b. When and how often.	Sections 2.6 and 2.7. Tissues were collected 24 h after plasmid injection. Injection volumes and sampling conditions are described in the Methods and figure legends.
		c. Where (including detail of any acclimatisation periods).	Sections 2.1, 2.4, 2.5, and 2.7. Experiments were performed under institutional animal experiment approval. Spleen, liver, and dorsa skin were used as target tissues.
		d. Why (provide rationale for procedures).	Introduction and Sections 2.6–2.8. The procedures were performed to evaluate how microdose jet input influences local tissue retention or penetration and functional
**Results**	10	For each experiment conducted, including independent replications, report:	
		a. Summary/descriptive statistics for each experimental group, with a measure of variability where applicable (e.g. mean and SD, or median and range).	Results Sections 3.1–3.5 and figure legends for Figs. 1–5 and Supplementary Fig. 2. Summary data are reported as mean ± SD where applicable, with individual values shown for the luciferase-expression datasets.
		b. If applicable, the effect size with a confidence interval.	Effect sizes with confidence intervals were not reported. Inferential statistical analyses and exact p-values are reported where applicable; the study was designed as an exploratory parameter-mapping study with relatively small group sizes.

The Recommended Set

These items complement the Essential 10 and add important context to the study. Reporting the items in both sets represents best practice.

Item		Recommendation	Section/line number, or reason for not reporting
**Abstract**	11	Provide an accurate summary of the research objectives, animal species, strain and sex, key methods, principal findings, and study conclusions.	Abstract. The revised abstract summarizes the
**Background**	12	a. Include sufficient scientific background to understand the rationale and context for the study, and explain the experimental approach.	Introduction. The background describes local nucleic acid delivery, needle-free jet injection, tissue mechanical heterogeneity, and the rationale for defining a tissue-dependent retention–expression window.
		b. Explain how the animal species and model used address the scientific objectives and, where appropriate, the relevance to human biology.	Introduction and Section 2.1. Mouse spleen, liver, and skin were selected as structurally and mechanically distinct target tissues to evaluate tissue-dependent retention and functional expression after microdose jet injection.
**Objectives**	13	Clearly describe the research question, research objectives and, where appropriate, specific hypotheses being tested.	End of Introduction. The objective was to deter
**Ethical statement**	14	Provide the name of the ethical review committee or equivalent that has approved the use of animals in this study, and any relevant licence or protocol numbers (if applicable). If ethical approval was not sought or granted, provide a justification.	Sections 2.1 and 7. All animal procedures were approved by the Animal Experiment Review Committee of the Animal Reproduction Research Institute, Ibaraki, Japan (animal experiment protocol approval No. 5-27; experimental protocol No. 20230802-04; experimental code No. MDD-M-T-ETC20021-24). Under the institutional animal experiment approval system used for this study, these approval and protocol identifiers were issued for the
**Housing and husbandry**	15	Provide details of housing and husbandry conditions, including any environmental enrichment.	Section 2.1. Animals were housed under stanc
**Animal care and monitoring**	16	a. Describe any interventions or steps taken in the experimental protocols to reduce pain, suffering and distress.	Section 2.1. All surgical exposure and injection procedures were performed under isoflurane anesthesia, and efforts were made to minimize pain and distress throughout the experiments.
		b. Report any expected or unexpected adverse events.	Section 2.1. No unexpected adverse events occurred during the experiments.
		c. Describe the humane endpoints established for the study, the signs that were monitored and the frequency of monitoring. If the study did not have humane endpoints, state this.	Section 2.1. Animals were monitored during and after procedures, and humane endpoints were applied if severe distress, marked bleeding, or abnormal recovery from anesthesia was observed. Animals were euthanized under deep isoflurane anesthesia
**Interpretation/ scientific implications**	17	a. Interpret the results, taking into account the study objectives and hypotheses, current theory and other relevant studies in the literature.	Discussion. The results are interpreted in relation to the study objective and the concept that functional carrier-free nucleic acid delivery depends on the interaction among physical jet input, tissue-specific retention behavior, and biological output rather than on
		b. Comment on the study limitations including potential sources of bias, limitations of the animal model, and imprecision associated with the results.	Discussion, final limitations paragraph. Limitations include the exploratory design and relatively small sample sizes, absence of a formal a priori sample-size calculation, representative rather than repeated jet-output measurements, dye-based retention
**Generalisability/ translation**	18	Comment on whether, and how, the findings of this study are likely to generalise to other species or experimental conditions, including any relevance to human biology (where appropriate).	Discussion. The findings are presented as a device-, payload-, and tissue-context-dependent framework rather than a universal delivery threshold. The retention–expression concept may help guide tissue-specific optimization of needle-free, carrier-free local nucleic acid delivery, but further studies across additional tissues, payloads, and delivery conditions are required
**Protocol registration**	19	Provide a statement indicating whether a protocol (including the research question, key design features, and analysis plan) was prepared before the study, and if and where this protocol was registered.	Section 2.9. A formal protocol was not registered.
**Data access**	20	Provide a statement describing if and where study data are available.	Data Availability Statement. The data that sup
**Declaration of interests**	21	a. Declare any potential conflicts of interest, including financial and non-financial. If none exist, this should be stated.	Declaration of Interest Statement. Kazuhiro Terai is affiliated with both Kumamoto University and Daicel Corporation. Hiromitsu Iga and Seiji Sakano are employees of Daicel Corporation, which develops the Actranza Lab jet injector used in this study. The other
		b. List all funding sources (including grant identifier) and the role of the funder(s) in the design, analysis and reporting of the study.	Funding. This research did not receive any specific grant from funding agencies in the public, commercial, or not-for-profit sectors.
