# Supplementary material for: Microdose jet injection reveals a tissue-dependent retention–expression window for carrier-free nucleic acid delivery
Source: Drug Deliv. 2026 Oct 30;33(1):2738976. doi: 10.1080/10717544.2026.2738976 (PMC13726303; doi:10.1080/10717544.2026.2738976)
Supplement: Supplementary file 2 [file IDRD_A_2738976_SM3102.docx]

**Supplementary Fig. 1. Extra-abdominal liver exposure and hepatic tissue sampling in mice.**

(A–D) Procedure for liver exposure and tissue collection. The central abdomen was shaved, a small incision was made, and gentle lateral compression was applied to expose the liver without apparent tissue injury. During injection, the container tip was placed lightly against the liver surface while maintaining stable contact. For sample collection, a hepatic tissue block of approximately 1 cm × 1 cm centered on the injection site was excised.

**Supplementary Fig. 2. Comparison of splenic luciferase expression following 1-µL administration by a 30-G Hamilton microsyringe (30G-HS) or Actranza Lab.**

Individual values and mean ± SD are shown on a logarithmic y-axis; n = 4 per group. **p* = 0.0148, two-sided Welch’s unpaired t-test.
