# Supplementary material for: Microdose jet injection reveals a tissue-dependent retention–expression window for carrier-free nucleic acid delivery
Source: Drug Deliv. 2026 Oct 30;33(1):2738976. doi: 10.1080/10717544.2026.2738976 (PMC13726303; doi:10.1080/10717544.2026.2738976)
Supplement: Supplementary file 3 [file IDRD_A_2738976_SM3104.docx]

Highlights

• Microdose jet injection reveals a tissue-dependent retention–expression window.
• Spleen and liver show progressive retention failure as jet input increases.
• Low-volume retained delivery yields high volume-normalized expression in parenchymal tissues.
• Skin supports broad local retention and a positive volume–expression relationship.
• Tissue-specific retention may guide optimization of carrier-free nucleic acid delivery.
